# Peripheral neuropathy associated with chronic lymphoproliferative disorders of natural killer cells (CLPD-NK): a case report and literature review

**DOI:** 10.1186/s12883-023-03310-7

**Published:** 2023-09-01

**Authors:** Hong An, Jiaxiang Guo, Hongliang Guo, Wenli Hu, Ming Lu

**Affiliations:** Department of Neurology, Beijng Chaoyang Hospital, Capital Medical University, No. 8 South Gongti Road, Chaoyang District, Beijing, 100020 China

**Keywords:** Peripheral neuropathy, CLPD-NK, CNKL, Chronic lymphoproliferative disorders of natural killer cells ; chronic natural killer cell lymphocytosis

## Abstract

**Background:**

Chronic lymphoproliferative disorders of natural killer cells (CLPD-NK) is a rare lymphoproliferative disease. Peripheral neuropathy is an unusual symptom of CLPD-NK. We report a case of peripheral neuropathy associated with CLPD-NK and perform a review of literatures.

**Case presentation:**

a 62-year‐old woman presented with progressive numbness and weakness in both extremities. Electrophysiological examinations indicated a sensorimotor polyneuropathy. Peripheral blood examination revealed that the number of white blood cells (WBC) and lymphocytes were significantly increased. Flow cytometry analysis identified that 84% of the lymphocytes are NK cells that mainly expressed CD56, combined with variable expression of CD16, CD2, CD7, CD94, granzyme B, perforin, and CD158 but negative for CD3. Sural nerve biopsy revealed that a plethora of NK cells infiltrated into nerve fascicles. On treatment with combined cyclophosphamide and corticosteroids, her symptoms rapidly improved. Moreover, the absolute lymphocyte count and its proportion recovered to normal range after 3 months’ treatment.

**Conclusion:**

To the best of our knowledge, this is the first case report of peripheral neuropathy associated with CLPD-NK from Chinese. This rare lymphoproliferative disease should be considered if peripheral neuropathy combines with increased WBC or lymphocytes. Immunosuppressive drugs are the major treatment and most patients can achieve a good prognosis.

## Background

Chronic lymphoproliferative disorders of natural killer (NK) cells (CLPD-NK), which is also known as chronic natural killer cell lymphocytosis (CNKL), is a rare chronic lymphoproliferative disease that is characterized by a persistent state of elevated NK cells in peripheral blood [1]. CLPD-NK belongs to large granular lymphocyte leukemia (LGLL) [2]. The estimated incidence of CLPD-NK ranges from 0.2 to 0.72 cases per 1 million individuals per year [3, 4]. The clinical manifestations of CLPD-NK are highly heterogeneous. About half of patients are asymptomatic at diagnosis, with NK lymphocytosis being the unique abnormality. Other symptomatic CLPD-NK patients present with fatigue and/or B symptoms, autoimmune-associated disease, splenomegaly, and recurrent infection related to mild or severe neutropenia. peripheral neuropathy is a rare symptom, as reported frequency of CLPD-NK onset with peripheral neuropathy is only 3% [5].

## Case presentation

On March 17, 2022, a 62-year‐old woman was admitted to our neurology unit with a 15- day history of progressive numbness accompanied with weakness in all four extremities. Fifteen days before admission, she experienced numbness in both hands and feet. Three days later, she felt painful in both lower limbs. These symptoms gradually progressed and she developed weakness in both lower limbs and walked unsteadily at admission. Neurological examinations revealed level V- muscular strength in both lower extremities, with absent deep tendon reflexes in all extremities and decreased sensation to a pinprick in distal portions of lower extremities. She did not have other symptoms or physical findings except neurological signs on admission. Eleven days after admission, she was almost unable to walk with severe burning pain in both lower extremities. Neurological examinations found that the muscular strength developed to level V- in both upper extremities and level II in both lower extremities. The patient has hypertension for 10 years (controlled with “nifedipine delayed-release tablet, 20 mg per day”) and negative family history.

**Fig. 1:**
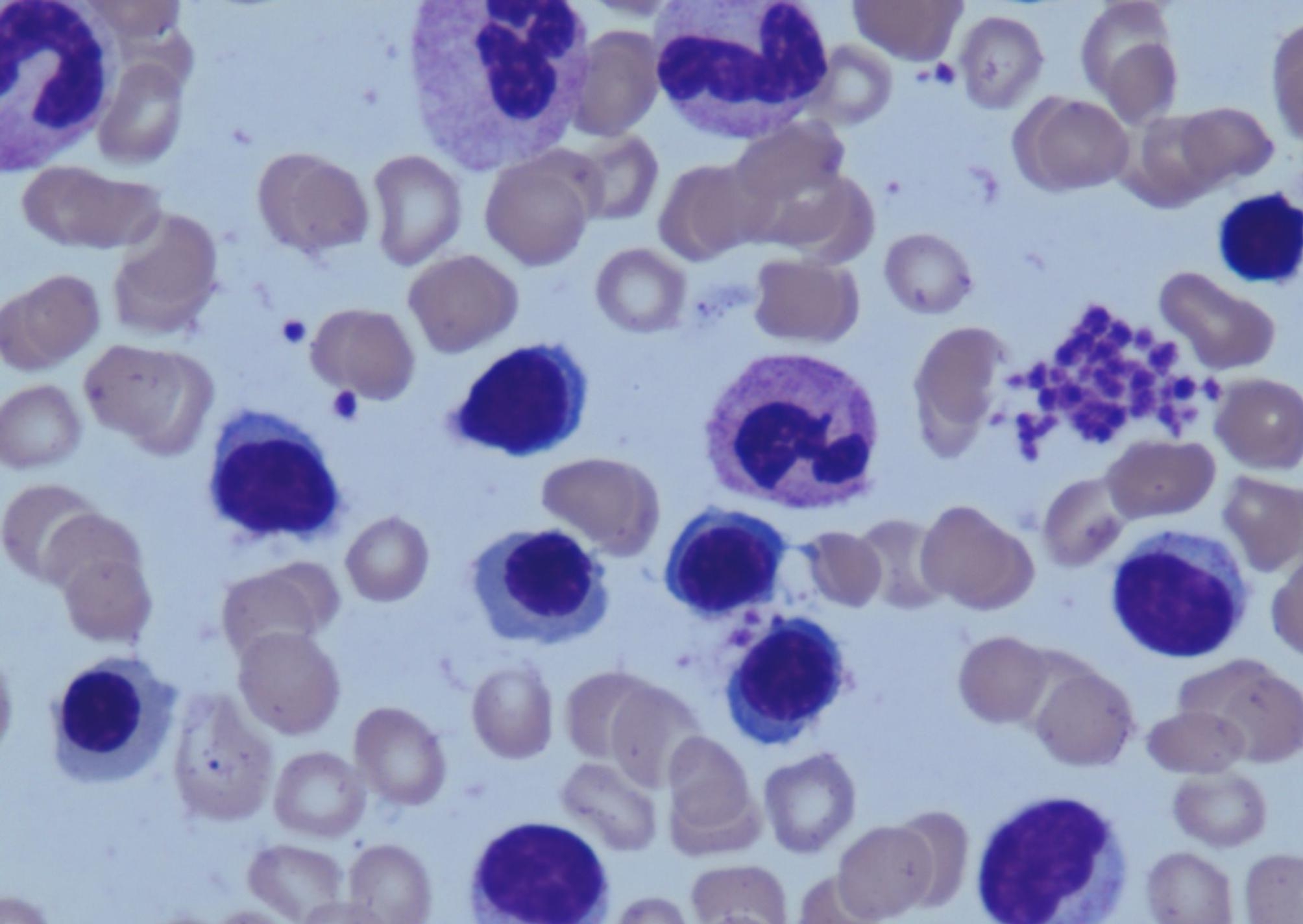
Peripheral blood smearing found large granular lymphocytes (Wright’s staining, ×1000)

**Fig. 2:**
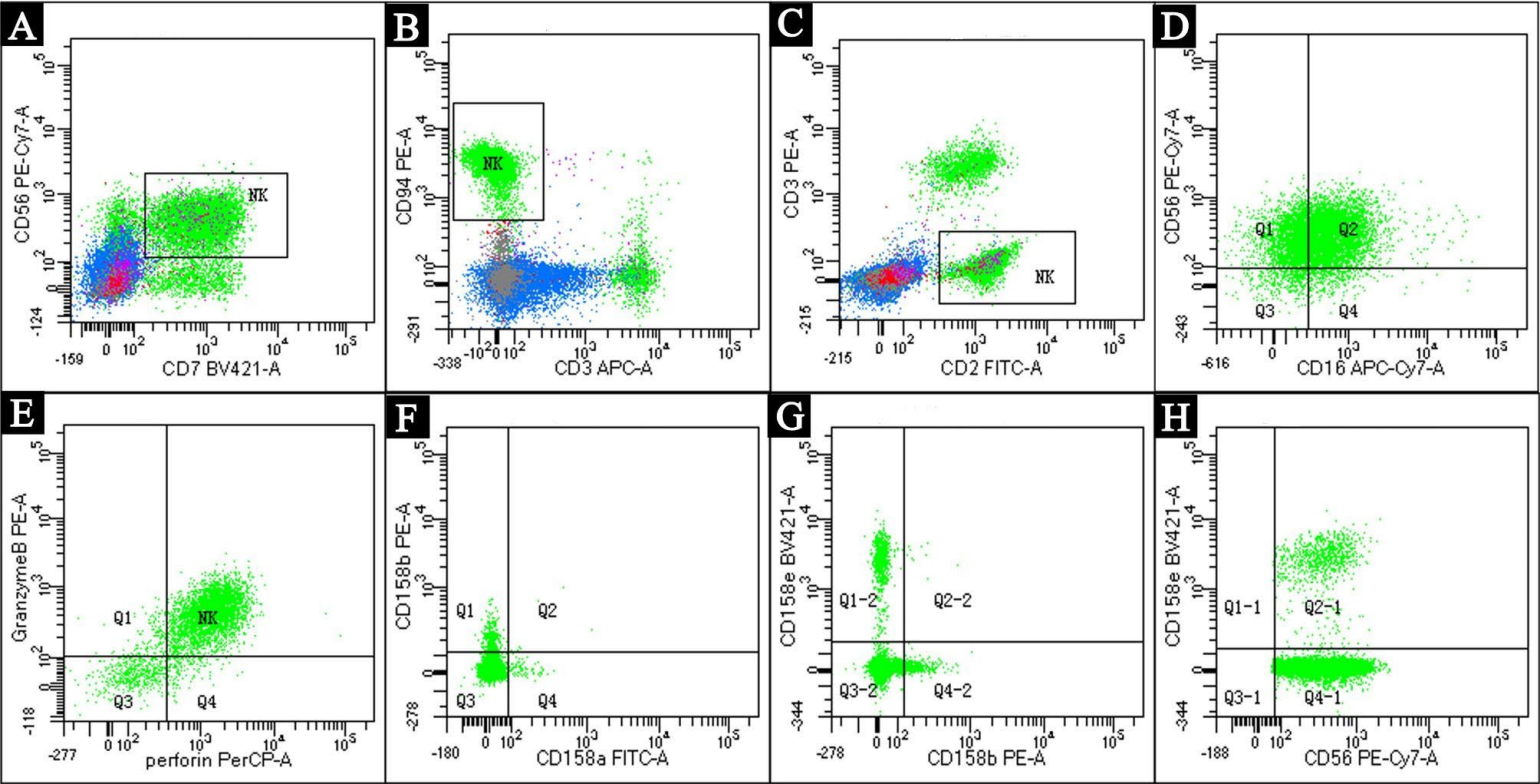
Bone marrow aspiration and lymphocytes analysis by flow cytometry revealed that the NK cells accounted for 84% of lymphocytes, which mainly expressed CD56 (A), CD7 **(A)**, CD94 **(B)**, and CD2 **(C)**, but negative for CD3 **(B&C)**. The NK cells also variablely expressed CD16 **(D)**, granzyme B **(E)**, perforin **(E)**, CD158a **(F)**, CD158b **(F&G)**, and CD158e **(G&H)**.

The peripheral white blood cell (WBC) count was 24.66 × 10^9^/L. Among which the absolute value and proportion of lymphocytes were 17.34 × 10^9^/L and 70.3%, respectively (Table 1). The red blood cell (RBC) count was 4.99*10^12^/L, hemoglobin was 157 g/L, and platelet count was 221 × 10^9^/L. Analysis of T cell antigen receptors and count of lymphocyte subsets showed a prominently increase of CD16^+^CD56^+^ cells (11,126, normal range 150–1100), indicating a NK cell immunophenotype. Peripheral blood smear observed large granular lymphocytes (LGLs), with round or reniform nucleus and an abundant cytoplasm that contained azurophilic granules (Fig. 1). Flow cytometry analysis, which performed on bone marrow specimens, revealed 84% of the lymphocytes are NK cells that mainly expressed CD56, combined with variable expression of CD16, CD2, CD7, CD94, granzyme B, perforin, and CD158 but negative for CD3 (Fig. 2). Lumbar puncture indicated normal cerebral spinal fluid (CSF) pressure (initial pressure was 180 mmH_2_O) with normal WBC count (2 × 10^6^/L, normal range: 0–8 × 10^6^/L) and protein level (35 mg/dL, normal range: 15-45 mg/dL). Blood antineutrophil cytoplasmic antibody, anti-nuclear antibodies, anticardiolipin antibody, and immunofixation electrophoresis were all negative.

**Table 1:** Blood routine examinations on admission and at follow-up

Blood routine examination	WBC (×10^9^/L)	ALC (×10^9^/L)	LYMR (%)
On admission	24.66	17.5	71
3 weeks after treatment	14.73	11.11	75.5
3 months after treatment	11.33	3.41	30.1

WBC: white blood cell, ALC: absolute lymphocyte count, LYMR: lymphocyte rate

**Fig. 3:**
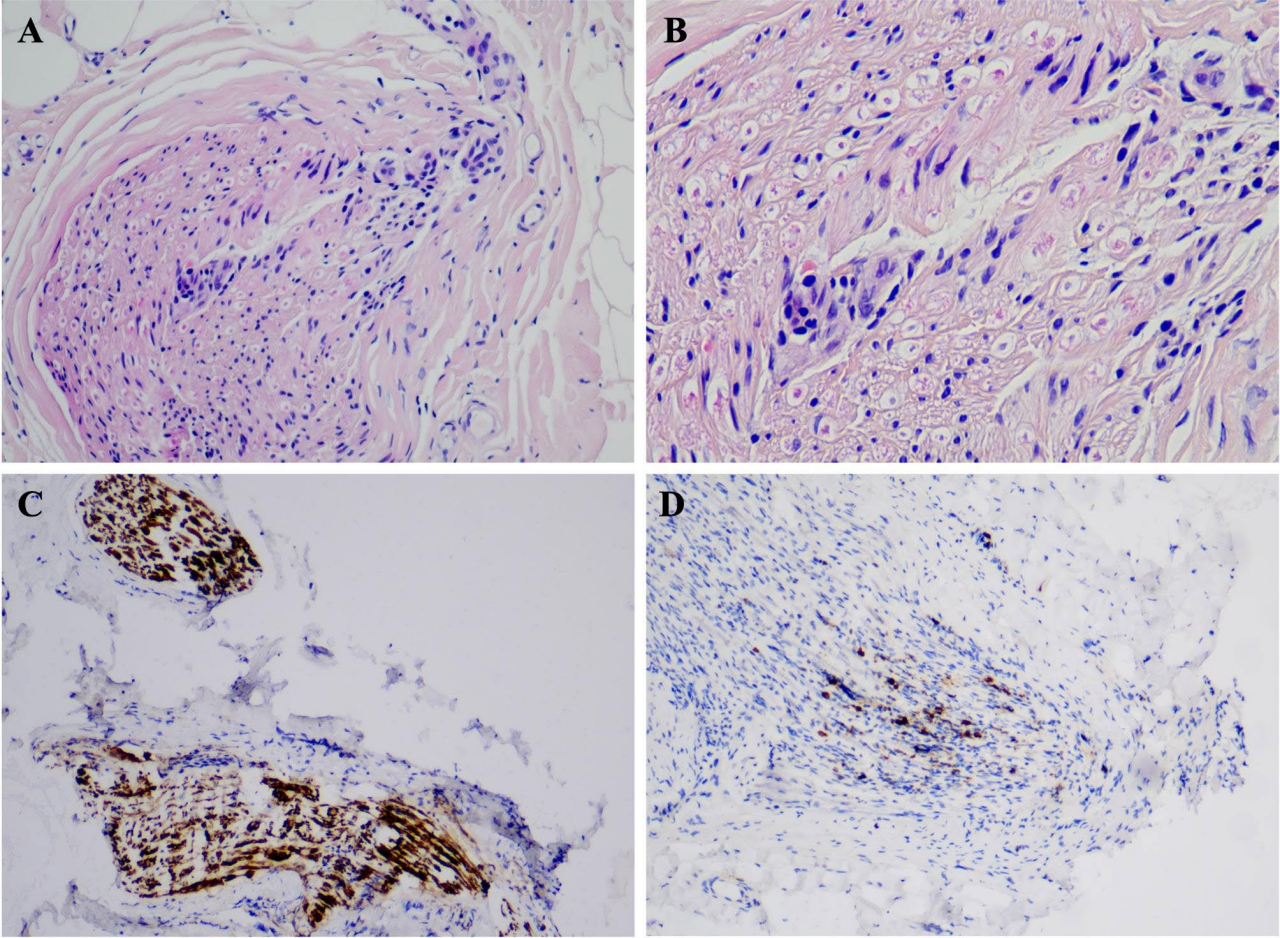
Sural nerve biopsy. HE staining demonstrated a plethora of lymphocytes that infiltrated into nerve fascicles **(A&B)**. Immunohistochemical staining and analysis demonstrated the major cells strongly expressed CD56 **(C)**, indicating NK cell immunotype. There was also a small number of CD3 positive cells, which might be T lymphocytes **(D)**

Nerve conduction study (NCS) demonstrated conduction blocks in left median nerve, left ulnar nerve, right tibial nerve, and right peroneal nerve, and reduced nerve conduction velocity in sensory nerve action potential of bilateral tibial nerves. F wave was unrecordable in left tibial nerve. H reflex was absent in bilateral tibial nerves. A needle electromyography revealed increased amplitude in bilateral tibialis anterior and left vastus medialis with spontaneous potentials in bilateral tibialis anterior. These electrophysiological findings indicated a sensorimotor polyneuropathy with predominant demyelinating. Left sural nerve biopsy was done. Hematoxylin-eosin staining revealed that a plethora of lymphocytes infiltrated into nerve fascicles, which was further being identified as NK cells by immunohistochemical method (Fig. 3). On treatment with methylprednisolone sodium succinate (120 mg, once per day, Intravenous) for 10 days followed by prednisone acetate (90 mg, once per day, Oral), cyclophosphamide (CTX, 50 mg, once per day, Oral), and pregabalin (150 mg, twice per day, Oral), her symptoms rapidly improved. The muscle power recovered to normal in both upper extremities and level IV in both lower extremities after 3 weeks’ treatment. The muscle power in lower extremities further recovered to normal at 3 months’ follow-up combined with normal deep tendon reflexes. Peripheral WBC count recovered to 14.73 × 10^9^/L after 3 weeks’ treatment and 11.33 × 10^9^/L after 3 months’ treatment. The absolute lymphocyte count and its proportion were 11.1 × 10^9^/L and 75.5% after 3 weeks’ treatment and recovered to normal range after 3 months’ treatment (Table 1).

## Discussion and conclusions

CLPD-NK is defined as a sustained proliferation of LGLs with NK cell immunophenotype for at least 6 months. The absolute NK cell count that required for the diagnosis is more than 0.5 × 10^9^/L [6, 7]. When NK cell count is less than 0.5 × 10^9^/L, bone marrow aspirate and/or biopsy with immunohistochemistry is mandatory for initial evaluation [8]. The pathogenesis of this disease is still incompletely understood. Previous studies have suggested that the disease may be initiated by immunological stimuli due to autoimmune disorders or chronic viral infections such as Epstein-barr virus (EBV) [9]. The autoantigens or viral peptides triggered abnormal amplification of NK cells, which was subsequently maintained by a proinflammatory microenviroment via constitutive activation of several pro-survival signaling pathways including JAK-STAT axis. Somatic mutations in STAT3 and STAT5b have been identified in approximately 8–30% of CLPD-NK patients [9]. However, since inducing STAT mutation is not enough to trigger LGLL in mice models [10], it does not play a causal role in development of the disease.

Peripheral neuropathy caused by CLPD-NK shows no specific manifestation on NCS. Patients can present a predominant demyelinating in the early stage but develop to a mixed axonal and demyelinating along with the course of the disease. Both motor and sensory nerves can be involved. Sural nerve biopsy is the golden standard for final diagnosis. Noguchi et al. [11]. first demonstrated that NK cells infiltrated into nerve fascicles in patient of CLPD-NK associated peripheral neuropathy. Using electron microscope, the authors observed that the infiltrated NK cells were close to myelinated fibers and showed breakdown of Schwann cell membrane, indicating NK cells might directly damage myelin and Schwann cells via a cytotoxic mechanism [11]. The mechanism may carry out by two ways. Firstly, NK cells contain granzyme B and perforin which can directly lysis myelin protein. Secondly, NK cells share a common antigenic determinant with peripheral nervous system, through which NK cells bind to Schwann cell surface leading to destabilization of myelin [11].

A literature review found 10 reported cases so far (Table 2). Median age at diagnosis was 63.5 (range 16–70) years old with no gender difference (5 male, 5 female). All patients presented with symptoms or signs of peripheral neuropathy such as paresthesia and limb weakness with reduced deep tendon reflexes, which were further demonstrated by electromyography. In addition, some of them were accompanied by a variety of other symptoms that has been reported in CLPD-NK patients [5], including fatigue, recurrent infection, hepatosplenomegaly, lymphadenopathy, skin lesions, and vasculitis. One case also manifested as abnormal sweating and postural hypotension indicating autonomic neuropathy. The WBC count was increased in 6 cases, within normal range in 2 cases, and not available in 2 cases. Of the two cases who showed normal WBC count, one had increased NK cells, and another one had atypical lymphocytes. Absolute lymphocyte count (ALC) or NK cell count was elevated in all available cases with increased percentage. CSF tests found NK cells in 2 cases, elevated WBC count in 1 case, and normal WBC count in 2 cases. Protein in CSF was elevated in 3 cases, and within normal range in 1 case. Sural nerve biopsy revealed inflammatory changes in 8 cases, 6 of which found lymphocytic infiltrates and the infiltrated lymphocytes were further demonstrated as NK cells in 5 cases. Nine of 10 cases received corticosteroids, 5 of which used prednisone, 2 cases used prednisolone, and another 2 cases did not provide detailed informations. One case received immune globulin. Five cases combined immunosuppressive therapy, including CTX (1 cases), MTX (2 cases), azathioprine (2 cases). One case received CHOP chemotherapy. Moreover, alemtuzumab was used in 1 case. All patients achieved relative good therapeutic responses except the 16-year-old patient from study by Wex et al. [12], whose symptoms worsened again after a transient improvement.

**Table 2:** Clinical characteristics of peripheral neuropathy associated with CLPD-NK

Reference	Age, years		Gender	Symptoms/signs	WBC (×10^9^/L)	ALC (×10^9^/L)	LYMR, (%)	EMG	WBC (CSF) (10^6^/L)	Protein (CSF) (g/L)	Sural nerve biopsy	Treatment	Prognosis
Wex et al.^7^	16	F		generalized muscle weakness, Lymphadenopathy, Allergic skin, Headaches, Recurrent infections, Delayed puberty	NA	32 (NK: 10.72)	NA	Decreased motor nerve conduction velocity	Increased NK cells	NA	Neuritic disease with perivascular infiltrates affecting small neural vessels	Prednisone	Achieved transient improvement
Boer et al.^26^	23		M	Lethargy, Anorexia, Headaches, Lower limb paraesthesia, Marked weight loss, Postural hypotension, Diarrhoea, Abnormal sweating, Mildly splenomegaly	16.25	NA	64	Reduced common peroneal distal motor amplitudes, mildly prolonged F-response latencies and absent H-reflexes. The sural amplitudes were at lower limit of normal. Suggesting a mild predominantly motor, axonal neuropathy or polyradiculopathy.	Found NK cells in CSF (no detail)	1.59	NA	MTX, CTX, teniposide prednisolone fludrocortisone	Achieved partial improvement of symptoms, left mild diarrhoea and postural hypotension, without obvious changes of blood white cell count and lymphocytes rate.
Rabbani et al.^1^	34		M	Skin lesions and peripheral neuropathy (bilateral extremity dyesthesias, loss of motor control and coordination) (Biopsy of skin lesions showed perivascular and perineural infiltration with atypical lymphoid cells)	5.5	1.88 (NK: 1.2)	NA (NK:64)	Sensorimotor peripheral neuropathy	NA	NA	Multifocal epineural perivascular lymphocytic infiltrates, focal areas of axonal loss consistent with ischaemic neuropathy	CHOP chemotherapy Prednisone	Improved
Rabbani et al.^1^	33		F	Diffuse macular erythematous skin lesions and peripheral neuropathy, (Biopsy of skin lesions revealed livedoid vasculitis)	11	7.32 (NK: 5.49)	NA (NK:75)	NA	NA	NA	NA	prednisone	Improved
Leitenberg et al.^27^	66		F	Upper and lower extremity paresthesias and progressive weakness	14.6	NA	67	Decreased motor nerve conduction velocities and prolonged F-wave latencies consistent with a demyelinating sensorimotor polyneuropathy	NA	NA	Inflammatory polyneuropathy with myelin loss and a mononuclear cell infiltrate	Azathioprine Prednisone	Achieved improvement in both neurologic symptoms and hematologic tests
Leitenberg et al.^27^	65		M	Slowly progressive tingling and weakness in the lower extremities, Mild splenomegaly	6.7	NA	6% lymphocytes;17% atypical lymphocytes	Decreased motor nerve conduction velocities and prolonged F-wave latencies consistent with a demyelinating sensorimotor polyneuropathy	NA	NA	Inflammatory polyneuropathy with myelin loss and NK cell infiltrate	Azathioprine Prednisone	Achieved improvement in both neurologic symptoms and hematologic tests
Noguchi et al.^15^	70		F	Progressive hypesthesia and weakness of upper and lower extremities and difficulty in walking, General malaise, Body weight loss, Hepatomegaly	17	NA	68	A mixed axonal and demyelinating neuropathy;	10	0.93	Infiltration of NK cells into the nerve fascicles, demyelinating changes combined with axonal degeneration	Prednisolone	Achieved both clinical and hematologic improvement
Chee et al.^20^	62		M	Livedo reticularis, Cutaneous polyarteritis nodosa, Peripheral neuropathy	14.6	8.91	82%	NA	NA	NA	Vasculitis, increased abnormal NK cell clone	Corticosteroids, MTX Alemtuzumab	Failed therapy with corticosteroids and MTX, but achieved both clinical and hematologic improvement by alemtuzumab
Richelli et al.^28^	65		M	Painful paresthesias and sensory loss in his feet, later involving volar surface of both hands, Gait disturbance, Weakness	NA	NA (NK:1.5)	NA	A diffuse demyelinating process with sighs of axonal degeneration especially at sural nerves.	3	1.53	Axonal degeneration and endoneurial mononuclear cell infiltrates, mainly composed of NK cells.	Immune globulin	Achieved both clinical and hematologic improvement
Sano et al.^29^	67		F	Progressive, asymmetric weakness and numbness in all four extremities	22.8	NA (NK:19.5)	94	Asymmetric demyelination in both motor and sensory nerves	1	0.27	Demyelination, NK cells infiltrated in the endoneurium.	Corticosteroids	Neurological, electrophysiological and hematological improvement

WBC: white blood cell, ALC: absolute lymphocyte count, LYMR: lymphocyte rate, NCS: nerve conduction study, CSF: cerebrospinal fluid, CHOP: cyclophosphamide, doxorubicin [adriamycin], vincristine, and prednisone, NA: not available

CLPD-NK should be distinguished with T-cell LGLL (T-LGLL), which shared similar clinical and biological features as well as treatment options [5]. Although peripheral neuropathy is a rare symptom, it can be observed in both CLPD-NK and T-LGLL^5^. Either axonal degeneration or demyelination can be observed in electrophysiologic tests in both subtypes. Therefore, it was quite challenging to distinguish these two subtypes based on symptoms or routine examinations. Experience from few case reports found that leukemic infiltration in nerve fascicles by sural nerve biopsy was only found in CLPD-NK but not T-LGLL [13, 14]. More importantly, immunophenotypical analysis by flow cytometry or immunohistochemical method are critical and useful tools for definite diagnosis [9]. T- lymphocytes display a CD3^+^, T-cell receptor (TCR)-αβ^+^, CD4^−^, CD8^+^phenotype, while NK cells show CD3^−^, TCR-αβ^−^, CD56^+^, CD16^+^, with variable CD57 expression [8, 9].

The optimal treatment of CLPD-NK has not yet been determined. Asymtomatic patients just need follow-up without treatment. Indications for therapy include: (1) severe neutropenia (absolute neutrophil count (ANC) < 0.5 × 10^9^/L); (2) symptomatic neutropenia (ANC between 0.5 × 10^9^/L to 1 × 10^9^/L associated with recurrent infection); (3) symptomatic or transfusion-dependent anemia; (4) symptomatic autoimmune diseases [6, 15]. The treatment of CLPD-NK is based on immunosuppressive therapy, as methotrexate (MTX) (10 mg/m^2^ per week) and CTX (50 to 100 mg per day) are the most common first-line treatments^9^. Therapeutic response was evaluated by clinical symptoms and hematologic tests after 4 to 6 months’ treatment. A hematologic complete response (CR) is defined as complete normalization of all affected lineages (i.e.,hemoglobin > 11 g/dL, platelet count > 150 × 10^9^/L, absolute neutrophil count > 1.5 × 10^9^/L) and peripheral blood LGLs count within the normal range [16]. Compared with MTX, CTX has the potential to eradicate cloning and seems to show better efficacy in controlling symptoms and cytopenia, which may provide sustained remission. However, if patients used CTX, the treatment should be maintained no more than 9–12 months to avoid severe complications [17]. Cyclosporine A (CyA) at a dosage of 3-5 mg/kg per day can be used as second-line therapy or first-line therapy for patients with severe anemia [9]. Studies have reported that the response rates of CTX, MTX, and CyA were similar with more than half patients showed CR, therefore, current treatment can be switched to another drug when the efficacy is not satisfactory [18]. Steroids including prednisone can be an adjunctive therapy to achieve a more rapid improvement, however, cannot be used separately^8^. If patients are refractory to all the above immunosuppressive treatments, purine analogs (including fludarabine, cladibrine, pentostatine and bendamustine) can be used as an alternative therapy [9]. Moreover, the efficacy of CD52 monoclonal antibody alemtuzumab has also been reported in very limited cases [19, 20]. However, the toxicities and availability of this drug obstruct its use in clinic. More importantly, a better mechanistic understanding will help to make future personalized therapies. For example, approaches that target JAK-STAT pathway have been suggested as promising salvage therapies in refractory cases but still limited in study stage so far [21]. The overall prognosis of CLPD-NK is benign, with a 10-year survival rate of 80% [ 22]. In rare cases, CLPD-NK may transform to ANKL or extranodal NK cell lymphoma with higher risk in EBV positive patients [23, 24].

In summary, we reported the first Chinese case of peripheral neuropathy associated with CLPD-NK. In addition, we systematically summarized the clinical, electrophysiological, and pathological characteristics of peripheral neuropathy associated with CLPD-NK. Our study will help clinicians to better understand this kind of rare but treatable disease. Timely diagnosis and treatment are critical for achieving a good prognosis. Further large studies are warrant for making standard and personalized therapeutic programs.

## Data Availability

The datasets used and/or analysed during the current study available from the corresponding author on reasonable request.
